# Prediction of autistic tendencies at 18 months of age via markerless video analysis of spontaneous body movements in 4-month-old infants

**DOI:** 10.1038/s41598-022-21308-y

**Published:** 2022-10-27

**Authors:** Hirokazu Doi, Naoya Iijima, Akira Furui, Zu Soh, Rikuya Yonei, Kazuyuki Shinohara, Mayuko Iriguchi, Koji Shimatani, Toshio Tsuji

**Affiliations:** 1 Department of Science and Engineering, Kokushikan University, Setagaya, Japan; 2 Graduate School of Engineering, Hiroshima University, Higashihiroshima, Japan; 3 Graduate School of Advanced Science and Engineering, Hiroshima University, Higashihiroshima, Japan; 4 School of Engineering, Hiroshima University, Higashihiroshima, Japan; 5 Graduate School of Biomedical Sciences, Nagasaki University, Nagasaki, Japan; 6 Faculty of Health and Welfare, Prefectural University of Hiroshima, Hiroshima, Japan

**Keywords:** Neurodevelopmental disorders, Autism spectrum disorders, Biomedical engineering, Computer science

## Abstract

Early intervention is now considered the core treatment strategy for autism spectrum disorders (ASD). Thus, it is of significant clinical importance to establish a screening tool for the early detection of ASD in infants. To achieve this goal, in a longitudinal design, we analyzed spontaneous bodily movements of 4-month-old infants from general population and assessed their ASD-like behaviors at 18 months of age. A total of 26 movement features were calculated from video-recorded bodily movements of infants at 4 months of age. Their risk of ASD was assessed at 18 months of age with the Modified Checklist for Autism in Toddlerhood, a widely used screening questionnaire. Infants at high risk for ASD at 18 months of age exhibited less rhythmic and weaker bodily movement patterns at 4 months of age than low-risk infants. When the observed bodily movement patterns were submitted to a machine learning-based analysis, linear and non-linear classifiers successfully predicted ASD-like behavior at 18 months of age based on the bodily movement patterns at 4 months of age, at the level acceptable for practical use. This study analyzed the relationship between spontaneous bodily movements at 4 months of age and the ASD risk at 18 months of age. Experimental results suggested the utility of the proposed method for the early screening of infants at risk for ASD. We revealed that the signs of ASD risk could be detected as early as 4 months after birth, by focusing on the infant’s spontaneous bodily movements.

## Introduction

Autism spectrum disorder (ASD) is a developmental disorder, typically characterised by a collection of symptoms, including repetitive behaviours, restricted interests, and poor social communication skills^1^. ASD is generally considered to have genetic underpinnings^2^. Recently, the prevalence of ASD has been increasing. This is partly owing to increased public awareness and changes in the ASD diagnostic criteria. However, a significant increase in the prevalence of ASD remains unexplained; hence, there is a pressing need for establishing reliable treatment strategies for ASD.

At this point, there is no definitive cure for ASD, but an increasing number of studies indicate the efficacy of early intervention for children with ASD^3,4^, which is manifested in improved prognosis and social adaptation. Thus, early detection of children at risk for ASD and early intervention are now considered to be a core treatment strategy for ASD^5^.

It is generally accepted that ASD can be diagnosed at around 3 years of age. However, a number of studies have indicated that early signs of ASD can be detected during infancy and toddlerhood^6–9^. For example, a retrospective study by Osterling *et al.* analyzed birth-day videos of ASD and typically developed (TD) children, and found that children later diagnosed with ASD showed lower social functioning compared with TD children^8^. Likewise, a prospective study by Elsabbagh *et al.* revealed that children with ASD show atypical electrophysiological activation to socially significant facial information (e.g. direct gaze), compared with their siblings without ASD and with TD children as young as 10 months of age^10^.

Previous findings pertaining to the early signs of ASD raise the possibility that children at risk for ASD, who have a high possibility of receiving diagnosis of ASD at later stages of development, can be screened during toddlerhood or, in some cases, during infancy. Early detection could lead to early intervention, potentially improving the prognosis for children with ASD and/or for those with sub-clinical-level symptoms. However, presently, there are no established tools for early detection of the ASD risk, and screening of children at risk for ASD requires time-consuming assessments and/or observations by teams of multidisciplinary professionals consisting of experienced clinicians and psychologists^11^.

Atypical motoric function is one of the most frequently reported signs of ASD during early development^12,13^. Many studies have reported various types of atypical bodily movement patterns, from infancy and toddlerhood, such as unbalanced posture and movement^14–16^, hypotonia^6^, spasticity in limb extremities^17^ and impairment in manual movement control^18^. These behavioural observations are in line with neuroimaging-based findings and histological studies on the brains of humans with ASD, in which atypical patterns have been reported regarding the morphology and function of motor-related neural regions, such as the basal ganglia and cerebellum^19,20^.

In studies on motor function during infancy, intensive attention has been paid to spontaneous movements in the supine position, which can be evaluated starting from the neonatal period. Neonates and infants exhibit a repertoire of bodily movement patterns in the supine position, which has been termed the general movement (GM). Prechtel *et al.* investigated the developmental trajectory of GM in preterm infants and proposed a framework for qualitative assessment of GM that can be used for predicting the risk of deficiency in higher-order brain functions, such as cerebral palsy^21,22^. Although the number is relatively small, several studies have also indicated the utility of GM for assessing the risk of psychiatric conditions^23,24^.

Considering the success of the GM assessment for predicting the risk of neurological and psychiatric conditions, together with the prevalence of atypical motoric development patterns in children with ASD, it seems feasible to establish an early screening method for objectively evaluating the risk of ASD based on the analysis of bodily motion patterns during early development. Several researchers have developed systems for automatic assessment of GM using image analysis techniques^25–27^. However, few studies addressed the possibility of the ASD risk evaluation. A notable exception is the MOVIDEA system^28,29^ which extracts kinematic features of infants’ motion using the automatic limb-tracking algorithm. Caruso *et al.* applied MOVIDEA to a large library of video recordings of infants’ spontaneous bodily movements, and found several motion features that discriminate high-risk infants later diagnosed with neurodevelopmental disorders from those without later diagnosis and typically developing infants^28^. The success of the MOVIDEA system indicates that semi-automatic analysis of bodily movement is a promising tool for the early detection of ASD.

**Figure 1:**
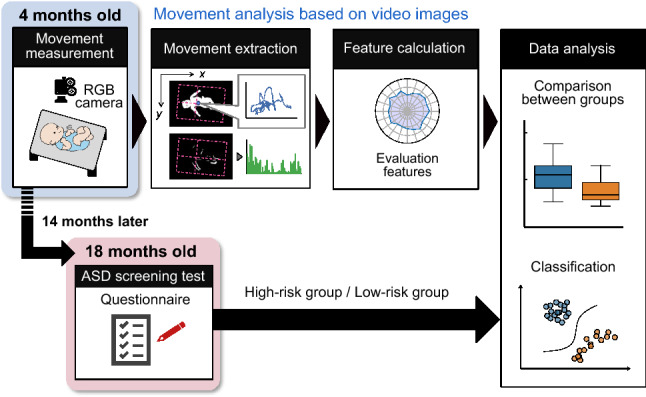
Schematic representation of infant video recording setting and the flow of information processing.

The primary goal of the present study was to examine whether bodily movement patterns during early infancy are predictive of the ASD risk, which becomes evident later in the development in a prospective design. To achieve this goal, we video-recorded spontaneous bodily movements in 4-month-old infants and quantified their features using a markerless system for infant movement evaluation, which is a modification of our conventional approach^30–33^. We focused on bodily movement patterns at 4-month-old mainly based on the following reasons. First, many motor-related regions including basal ganglia and brain stem, where the central pattern generator (CPG) is located, experience steady growth in its metabolism ^34^ and morphology^35^ during 3–6 months after birth. Thus, bodily movement features at 4-month-old can be a good indicator of cortical and subcortical development. Second, several studies have revealed that posture and bodily movement at this stage of development are predictive of later cognitive development well into school age^36^ (for a review, see Einspieler *et al.*^21^). Classifiers were trained to discriminate the ASD risk in 18-month-old infants, based on their bodily movement patterns at 4 months of age. The design of the current study is schematically shown in Fig. 1. In contrast to some of the other proposed methods of automated infant movement analysis, our system does not require special equipment or attachment of sensors to the body surface; thus, it can be easily implemented in clinical settings.

The risk of ASD in 18-month-old infants was evaluated using the Modified Checklist for Autism in Toddlerhood (M-CHAT)^37,38^. The M-CHAT is a questionnaire widely used for early screening of the ASD risk, and is reported to be applicable to toddlers as young as 18 months of age. Although the sensitivity of the M-CHAT method is not very high (Sensitivity = 0.550, Specificity = 0.961)^37^, it captures early signs of atypical development broadly in many domains, such as social communication and sensory processing.

## Methods

### Participants

Sixty-two 4-month-olds and their mothers participated in video recordings of bodily movement patterns in the present study as a part of the birth cohort study, after the caregivers gave their written informed consents. All infants were full-term births, weighed between 2288 and 3932 g (only three infants had low birth weight, i.e., less than 2500 g), and no intrauterine growth restrictions were observed. We used all the data available at the point of analysis without determining the sample size a priori, because the number of infants evaluated to be at high risk for ASD was predicted to be quite small. The mother-infant pairs were recruited in Nagasaki City, a rural city on Kyushu Island in Japan, through fliers or in-person advertisement in obstetrics and gynaecology clinics. The protocol of this study was reviewed and approved by the ethics committee of the Graduate School of Biomedical Sciences at Nagasaki University (Registration Numbers: 14050205-2 and 10093099) and the Graduate School of Engineering at Hiroshima University (Registration Number: E-1150-1). All methods were performed in accordance with the relevant guidelines and regulations.

### Procedure

Mother-infant pairs participated in the present study at two time points, i.e. 4 and 18 months after birth. At 4 months of age, the subjects visited our laboratory at Nagasaki University. At this point, we video-recorded the bodily movements of infants while lying in the supine position, for later analysis. Meanwhile, mothers completed several questionnaires regarding their mental state and the development of their infants. The questionnaires at 4 months after birth included Japanese translation of CESD^39^, EPDS^40^, and Bonding scale. Infant’s development was assessed by Kinder Infant Development Scale (KIDS)^41^. We sent out questionnaires, including M-CHAT, CESD, Bonding scale and KIDS, about a week before each infant reached the age of 18 months after birth, and asked the mothers to send back the questionnaires after answering them fully.

#### Bodily movement recording

Each infant subject was laid on a black mat, in a cream-white wooden crib. The infant’s body, except for the head, arms, and limbs, was covered in a white wrap, for increasing the contrast between the infant’s body and background. A video camera was affixed to a silver stainless bar arching over the crib, and was directed downward toward the infant, as shown in the leftmost panel of Fig. 1. The mother was not present during the video recording. The frame rate and resolution of the video recordings were $F_\mathrm {s} = 30$ fps and $W \times H = 720 \times 480$ pixels, respectively. The infant movements were recorded continuously. The average ± SD of the length of the recorded videos was 800.93 ± 280.83 s (24,028 ± 8,425 frames), ranging 320–1,698 s (9,600–50,940 frames). Background images were taken before or after the video measurements and used for image processing.

#### Self-administered questionnaires

We asked the mothers to complete self-administered questionnaires at three points. The first batch of questionnaires was administered at the time of enrolment. The questionnaires asked for demographic information regarding her and her husband’s age, socio-economic status, and medical history. At 4 months of age, information about the mother’s mental state and infant’s developmental state was collected at the lab; the results of this analysis will be reported elsewhere, but these descriptive statistics are presented in Supplementary Table S1. At 18 months of age, we mailed questionnaires, including the M-CHAT. The M-CHAT included 23 items of Yes/No questions, asking whether a child exhibited developmental delays in socio-cognitive functions (see Supplementary Table S2). The number of failed items exceeding the cutoff criterion is judged to indicate a high risk of ASD. The M-CHAT also included items about atypical behaviors often observed in ASD, such as hypersensitivity to sensory stimulation. In the Japanese version of M-CHAT, a subset of ten items (Items 2, 6, 7, 9, 13, 14, 15, 20, 21, and 23) was defined as critical^42^. The cutoff criterion was set to one of the critical items or any three items.

### Analysis

#### Bodily movement analysis

**Figure 2:**
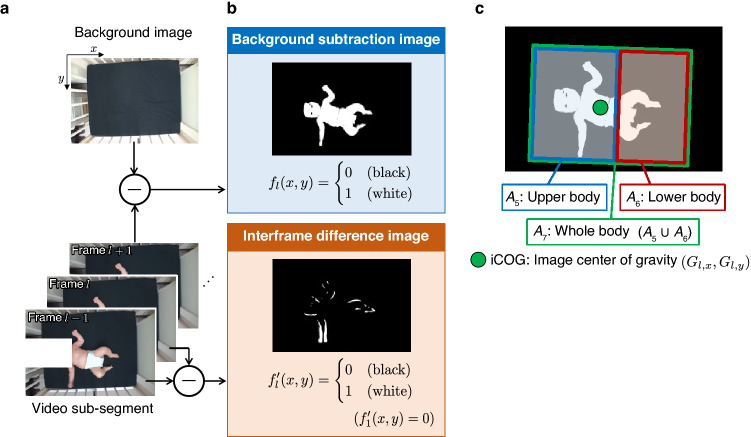
Image processing procedure. (**a**) Recorded videos were divided into video sub-segments. Background image was also captured for each infant. (**b**) Background subtraction images and interframe difference images were then calculated. (**c**) Several body regions were defined based on the rectangle surrounding the infant’s body. In this paper, three body regions, upper body ($A_5$), lower body ($A_6$), and whole body ($A_7$), were used for analysis.

In the initial stage of processing, the recorded videos were divided into *S* video sub-segments by deleting frames that contained the infants’ sleep, crying, and occurrence of external stimulation, including any social interactions with the caregivers or experimenter (Fig. 2a). To extract the image of the infant’s body as the foreground, we calculated the background subtraction image $f_l (x,y)$ $(x = 1,2,\ldots ,W;\ y = 1,2,\ldots ,H)$, in which the pixel value was 1 (white) if the pixel value of the brightness image became greater than or equal to the threshold $T = 80$; the value was 0 (black) in all other cases (Fig. 2b). Note that *l* ($l = 1,2,\ldots ,L_s$) represents the number of frames, and $L_s$ is the frame length of the *s*-th video sub-segment. The coordinates in the image are represented as (*x*, *y*), which correspond to the vertical and horizontal directions of the body, respectively. To extract the movement information, the interframe difference image $f'_l (x,y)$ was obtained by frame-by-frame subtraction (Fig. 2b). Note that $f'_1 (x,y) = 0$. To reduce the salt-and-pepper noise caused by changes in light and wrinkles on the sheets included in these difference images, we performed image erosion, followed by image dilation. Each of these processes was repeated thrice.

To evaluate the motion of each body region, the video images were divided into several regions using background subtraction images (Fig. 2c). First, the outer circumference of the infant’s trunk within each frame was approximated using an ellipsoid. Then, a rectangle was defined such that the rectangle contained an ellipsoid within it with a margin^31^. Based on the rectangle defined around the infant body in each frame, seven bodily regions, $A_k$ $(k = 1,2,\ldots ,7)$, were defined. Regions $A_{1}$–$A_{4}$ represented the upper right, upper left, lower right, and lower left quadrants, respectively. Regions $A_{5}$ and $A_6$ were defined as $A_1 \cup A_2$ and $A_3 \cup A_4$, respectively, thus representing the upper and lower body. Finally, the whole body, $A_7$, was defined as $A_5 \cup A_6$.

From the background subtraction image and interframe difference image, body posture, ${^{(A_k)}}P_l$, and body movement, ${^{(A_k)}}M_l$, in each area $A_k$ in the *l*-th frame ($l \le L_s$) were extracted, based on the following equations:

1 $$\begin{aligned} ^{(A_{k})}P_{l}&= {\sum _{(x, y) \in {A_{k}}}}{{f_{l}(x, y)}}, \end{aligned}$$

2 $$\begin{aligned} ^{(A_{k})}M_{l}&= \frac{1}{^{(A_{7})}P^\mathrm{{avg}}}{\sum _{(x, y) \in {A_{k}}}}{{f^{\prime }_{l}(x, y)}}, \end{aligned}$$

where ${}^{(A_{7})}P^\mathrm{{avg}}$ represents the average of the maximal values of ${{^{(A_7)}}P}_l$ up to the *E*-th frame of the first $L_e$ $(E \le L_e)$ frames of the first video sub-segment. When ${{^{(A_k)}}M}_l$ exceeded the threshold value of $M_{\mathrm {th}}$, the *l*-th frame was judged to contain a bodily movement in area $A_k$. We hereinafter refer to a frame that contains a bodily movement in area $A_k$ as “a frame with a bodily movement.”

The image center of gravity (iCOG), $\left( G_{l,x}, G_{l, y} \right)$, which represents the coordinates of the body center at the *l*-th frame, were calculated by the following equations:

3 $$\begin{aligned} G_{l, x}&= \frac{1}{^{(A_{7})}P_{l}}{\sum _{(x, y) \in {A_{7}}}}{{xf_{l}(x, y)}}, \end{aligned}$$

4 $$\begin{aligned} G_{l, y}&= \frac{1}{^{(A_{7})}P_{l}}{\sum _{(x, y) \in {A_{7}}}}{{yf_{l}(x, y)}}. \end{aligned}$$

The velocity of the body center, $(G_{l,x}^\mathrm {v}, G_{l,y}^\mathrm {v})$, was calculated as the frame-by-frame difference of the iCOG coordinates at the *l*-th frame:

5 $$\begin{aligned} G^\mathrm{{v}}_{l, j} = \frac{F_\mathrm {s} (G_{l, j}-G_{l-1, j})}{\sqrt{^{(A_{7})}P^\mathrm{{avg}}}}, \end{aligned}$$

where $j \in \{x, y\}$ and $(G_{1,x}^\mathrm {v}, G_{1,y}^\mathrm {v}) = (0, 0)$. The fluctuation of the body center, $(G_{l,x}^\mathrm {d},\ G_{l,y}^\mathrm {d})$, was calculated based on the average of the iCOG coordinates, $\left( G_x^\mathrm {ave},\ G_y^\mathrm {ave}\right)$, over the first $L_e$ frames of the first video sub-segment, by the following equation:

6 $$\begin{aligned} G^\mathrm{{d}}_{l, j} = \frac{G_{l, j}-G^\mathrm{{avg}}_{j}}{\sqrt{^{(A_{7})}P^\mathrm{{avg}}}}. \end{aligned}$$

To reduce noisy components involved in the body movement ${}^{(A_k)}M_l$ and body center velocity $G^\mathrm {v}_{l,j}$, we smoothed these values using second-order Butterworth low-pass filters. The cutoff frequencies for ${}^{(A_k)}M_l$ and $G^\mathrm {v}_{l,j}$ were set to 10 Hz and 5 Hz, respectively.

Based on the bodily movement data within each quadrant of the entire rectangle, we calculated 26 bodily movement features (Table 1), including 23 features that are part of the evaluation features proposed by^31^ and three features newly defined in this paper. The detailed definitions of these bodily movement features are explained in the Supplementary Materials. We also calculated the whole-body movement frequency, $^{(A_7)}I_1$, to analyze infants who exercised for more than a certain amount of time within the analysis segment.

**Table 1:** Summary of bodily movement features.

Category	Feature	Description
Movement magnitude	${^{(A_5)}I_1}$, ${^{(A_6)}I_1}$	Movement frequency
	${^{(A_5)}I_2}$, ${^{(A_6)}I_2}$	Movement strength
	${^{(A_5)}I_3}$, ${^{(A_6)}I_3}$	Movement count
Movement balance	${^{(A_5,\; A_6)}I_4}$	Ratio between ${^{(A_5)}I_1}$ (upper body) and ${^{(A_6)}I_1}$ (lower body)
	${^{(A_5,\; A_6)}I_5}$	Ratio between ${^{(A_5)}I_2}$ (upper body) and ${^{(A_6)}I_2}$ (lower body)
	${^{(A_5,\; A_6)}I_6}$	Movement coordination between upper body and lower body
Movement rhythm	${^{(A_5)}I_7}$, ${^{(A_6)}I_7}$	Central frequency of the motor alteration $^{(A_k)}M$
	${^{(A_5)}I_8}$, ${^{(A_6)}I_8}$	Second moments around ${^{(A_5)}I_{7}}$ and ${^{(A_6)}I_7}$
	$^{(A_7)}I_{9_{x}}$, ${^{(A_7)}I_{9_{y}}}$	Central frequency of the body center velocities $(G^\mathrm{{v}}_{x},~G^\mathrm{{v}}_{y})$
	$^{(A_7)}I_{10_{x}}$, $^{(A_7)}I_{10_{y}}$	Second moments around $^{(A_7)}I_{9_{x}}$ and ${^{(A_7)}I_{9_{y}}}$
	${^{(A_7)}I_{11_{x}}}$, $^{(A_7)}I_{11_{y}}$	Central frequency of the body center fluctuations $(G^\mathrm{{d}}_{x},~G^\mathrm{{d}}_{y})$
	$^{(A_7)}I_{12_{x}}$, $^{(A_7)}I_{12_{y}}$	Second moments around ${^{(A_7)}I_{11_{x}}}$ and $^{(A_7)}I_{11_{y}}$
Body center movements	$^{(A_7)}I_{13_{x}}$, $^{(A_7)}I_{13_{y}}$	Variations in the body center velocities
	$^{(A_7)}I_{14_{x}}$, $^{(A_7)}I_{14_{y}}$	Standard deviations of the body center fluctuations$^\dagger$
	$^{(A_7)}I_{15}$	Closed area in the outermost circumference of the body center excursion$^\dagger$

$A_5$: upper body, $A_6$: lower body, and $A_7$: whole body.

$^\dagger$Features newly introduced in this paper

#### Feature selection

We first selected the features that are useful for classifying infants into low and high ASD risk groups. Low and high-risk infants were defined according to MCHAT scores; those who scored below the cutoff were defined as the low-risk group and vice versa. In the first stage of feature selection, we tested group differences on 26 features between infants with low and high ASD risk, using the Brunner–Munzel test^43^ (significance level: 5%), which is a nonparametric statistical test for testing the equality of distributions of two unpaired samples. This test does not assume equal variances of the two samples.

Features that showed significant group differences at the 10% significance threshold were retained for further analyzes. This lenient threshold was adopted because we wished to retain as many informative features as possible for later classification using machine learning algorithms. In the second stage of feature selection, the correlation coefficients were computed for every pair of retained features. Pairs of features with correlation coefficients $\ge 0.7$ were lumped into a single feature.

#### Group classification using machine learning

We examined whether bodily movements contain sufficient information for discriminating infants with high ASD risk from those with low ASD risk, using several machine learning methods. Four classifiers were trained for predicting whether an infant belonged to the high-risk group based on the selected bodily movement features, using supervised learning. The four machine learning algorithms included two linear classifiers, i.e. the linear discriminant analysis (LDA) and the logistic regression (LR), and two non-linear classifiers, i.e. the multi-layered perceptron (MLP) and the log-linearised Gaussian mixture network (LLGMN)^44^. The MLP included one hidden layer, the activation function of which was a rectified linear unit.

The performance of the four classifiers was cross-validated using the leave-one-out procedure. In the leave-one-out approach, the data of one of the participants were treated as unknown test data, while the data of the remaining participants were used for training the corresponding classifier. This cycle was repeated until the data from every participant served as the test data. The MLP and LLGMN each included one hyperparameter, i.e. the number of hidden layers and the number of components, respectively. These hyperparameters were tuned using a nested leave-one-out procedure^45^.

Fisher’s exact test was performed for testing associations between the classifications made by each of the classifiers used in this study and grouping by the M-CHAT scores. Yule’s correlation coefficient (Yule’s *Q*) was also calculated, for confirming the strength of each association. The performances of the classifiers were compared in terms of the sensitivity, the specificity, the F1 score, the area under curve of receiver-operator-characteristic curve (AUC-ROC), and the area under the precision-recall curve (AUC-PR) as performance indicators.

## Results

Of the 62 mother-infant pairs who participated in the video-recording sessions at 4 months of age, 58 mothers returned the M-CHAT questionnaire at 18 months of age. Due to a small number of mothers who failed to return the questionnaires ($n = 4$), we did not formally test the existence of response bias. Among these 58 mother-infant pairs, bodily movement analysis was performed for 41 infants (27 males). Video recordings from 17 infants were discarded owing to their short length ($\le 3$ min; $n = 13$) and low frequency ($^{(A_7)}I_1 \le 10\%$; $n = 4$) of bodily movement in them. The reason for the former criterion is that the infant movement analysis system^31^ requires at least 3 min of video segment for feature calculation. The latter criterion was set because the infants whose whole-body movement frequency was less than 10% of the video segment length (i.e., less than 18 seconds out of 3 min) had difficulty with adequate movement assessment. Although three infants with low birth weight (2288, 2452, and 2482 g) were included in these 41 infants, all three were in the ASD low-risk group.

There were no significant differences between these 41 mother-infant pairs and 21 pairs whose data were discarded in terms of the days after birth ($W = 1.439$, $p = 0.156$), the weight at birth ($W = 1.227$, $p = 0.227$), the gestational age at birth ($W = 1.032$, $p = 0.308$), and mother’s age at birth ($W = -1.6$, $p = 0.119$). The distributions of high-and low-risk children did not differ between 41 mother-infant pairs and 17 mother-infant pairs whose M-CHAT data were available but were discarded from the final analysis ($p = 0.715$); this conclusion was reached based on Fisher’s exact test. The distributions of M-CHAT scores (total number of failed items) also did not differ significantly between these 41 pairs (0 for 23 pairs, 1 for 14 pairs, 2 for two pairs, and 3 for one pair) and 17 pairs (0 for 11 pairs, 1 for three pairs, 2 for two pairs, and 3 for one pair) based on Fisher’s exact test ($p = 0.3526$). Thus, although the data attrition rate was relatively high, there were no signs of selection bias.

Among the 41 infants, seven were evaluated to be at high risk for ASD (the high-risk group). In the high-risk group ($n=7$), the number of failed items was 1 for three infants, 2 for two infants, and 3 for two infants; in the low-risk group ($n=34$), 0 for 23 infants and 1 for 11 infants. The age-in-days on the day of video recording, weights at birth, the gestational ages of the infants, and the mothers’ ages, for the high-and low-risk groups, are summarized in Table 2. The Brunner–Munzel test did not reveal significant group differences either in age ($W = 0.067$, $p = 0.95$), weight at birth ($W = -1.400$, $p = 0.18$), gestational age ($W = 0.214$, $p = 0.835$), or mother’s age at birth ($W = 1.172$, $p = 0.265$).

**Table 2:** Background information of infants and mothers.

Category	Group		*W*	*p*-value
	High risk	Low risk		
Days after birth at recording (days)	128.6 (10.4)	125.9 (5.7)	0.067	0.949
Weight at birth (g)	2964.0 (221.7)	3121.2 (383.1)	$-$1.400	0.179
Gestational age (days)	278.9 (7.8)	278.1 (7.9)	0.214	0.835
Mother’s age at birth (years)	31.3 (3.6)	33.4 (4.9)	$-$1.172	0.265

In the parenthesis are the standard deviations.

### Feature selection

**Figure 3:**
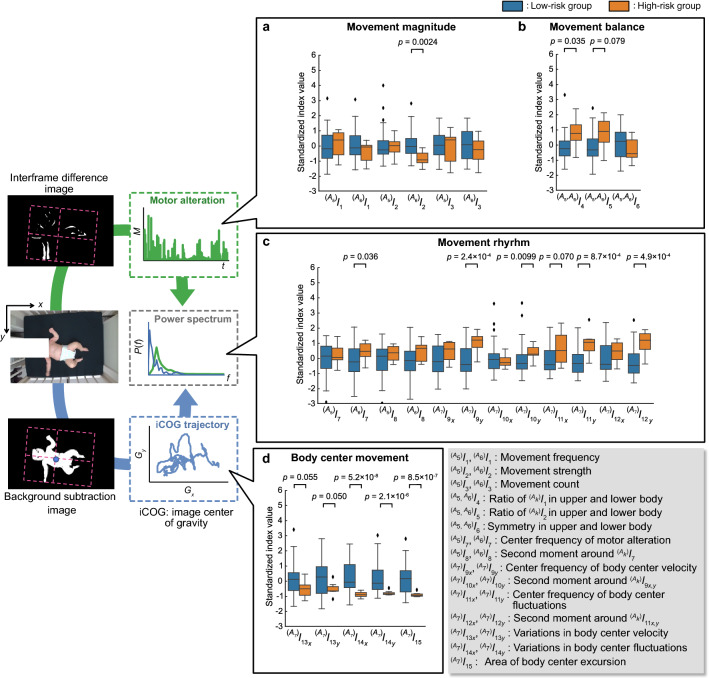
Boxplots of calculated features. (**a**) Movement magnitude. (**b**) Movement balance. (**c**) Movement rhythm. (**d**) Movement of the body center (iCOG movement) in each group. Brunner–Munzel test was used to compare the features between high and low ASD risk groups, and the *p*-values that resulted in $p < 0.1$ are shown.

**Figure 4:**
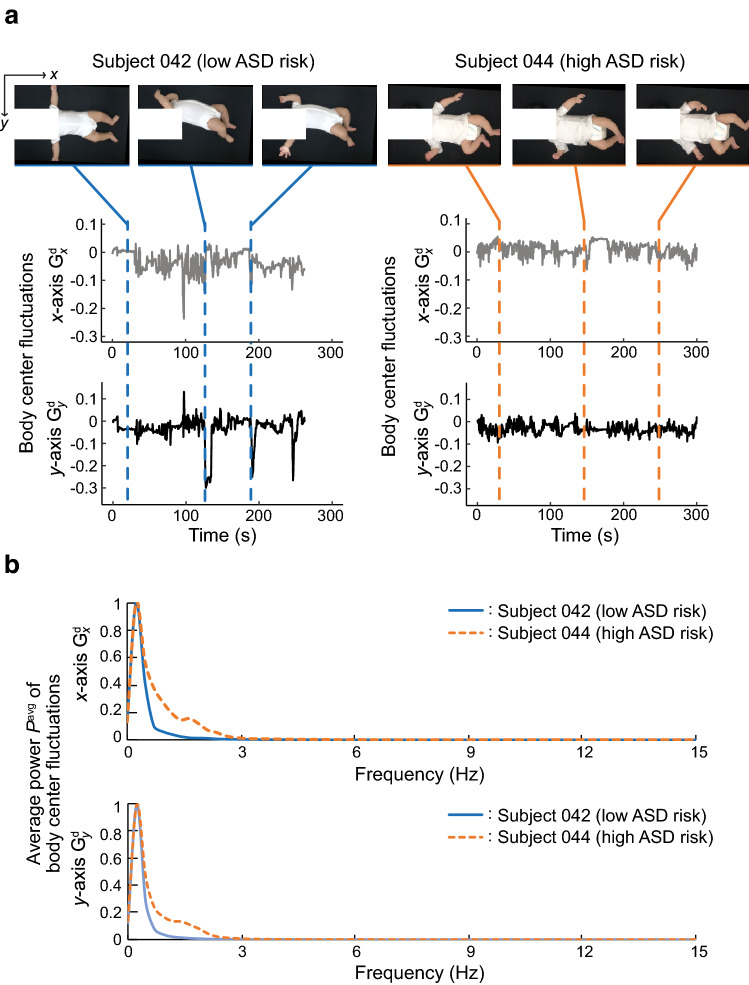
Examples of the calculated features. (**a**) Temporal course of body center fluctuation. (**b**) Averaged power spectrum densities of body center fluctuations. Each density is normalized such that the maximum value is equal to 1.

The boxplots of each bodily movement feature extracted from video recordings are shown in Fig. 3 for the low- and high-risk groups. As can be seen from Fig. 3, infants in the high-risk group exhibited significantly lower movement strength in their lower limbs (${^{(A_6)}}I_2$; $W = -4.10$; $p = 0.0023$) and lower balance of movement between their upper and lower limbs (${^{(A_5, A_6)}}I_4$; $W = 2.56$, $p = 0.035$). In the domain of movement rhythmicity, infants in the high-risk group exhibited higher central frequencies and larger second moments around the central frequency in the time series of the body center velocity, and a fluctuation along the left-right axis (${^{(A_7)}}I_{9_y}$, $W = 4.94$, $p < 0.001$; ${^{(A_7)}}I_{10_y}$ $W = 2.89$, $p = 0.0099$; ${^{(A_7)}}I_{11_y}$, $W = 4.17$, $p < 0.001$; ${^{(A_7)}}I_{12_y}$, $W = 4.76$, $p < 0.001$). Examples of the temporal course of body center fluctuation and the distribution of frequency power are shown for low- and high-risk infants in Fig. 4.

The standard deviation of the body center velocity and the fluctuation along the y-axis (${^{(A_7)}}I_{13_y}$, $W= -2.03$, $p= 0.0501$; ${^{(A_7)}}I_{14_y}$, $W =-5.59$, $p< 0.001$), and the area covered by the trajectory of the body center excursion (${^{(A_7)}}I_{15}$, $W = -5.96$, $p<0.001$) were significantly smaller in the high-risk group than in the low-risk group.

There were significant ($p < 0.05$) and marginally significant ($p < 0.10$) group differences across the 14 bodily movement features. For each pair out of the 14 features, we computed the correlation coefficient. Correlational coefficients between ${^{(A_7)}}I_{9_y}, {^{(A_7)}}I_{12_y}$, and ${^{(A_7)}}I_{11_y}$ and between ${^{(A_7)}}I_{11_y}$ and ${^{(A_7)}}I_{11_x}$ were above 0.7. Therefore, these four features were averaged into a single feature. The correlation coefficients between ${^{(A_7)}}I_{13_x}$ and ${^{(A_7)}}I_{13_y}$, ${^{(A_7)}}I_{13_x}$ and ${^{(A_7)}}I_{15}$, ${^{(A_7)}}I_{13_y}$ and ${^{(A_7)}}I_{15}$, ${^{(A_7)}}I_{14_x}$ and ${^{(A_7)}}I_{15}$, and ${^{(A_7)}}I_{14_y}$ and ${^{(A_7)}}I_{15}$ were all above 0.7. Thus, these five features were compressed into a single feature as well. After the compression, none of the absolute values of the pairwise correlation coefficients was above 0.7.

To evaluate the reliability of the proposed method from a different perspective, in addition to comparing high- and low-risk ASD groups based on cutoff criterion, we also analyzed the relationship between ordinal scores on the M-CHAT and behavioral characteristics (see Supplementary Materials). The results revealed that the important movement features were nearly identical whether the groups were compared dichotomously based on cutoff criterion or the M-CHAT score itself was regressed.

### Group classification

**Figure 5:**
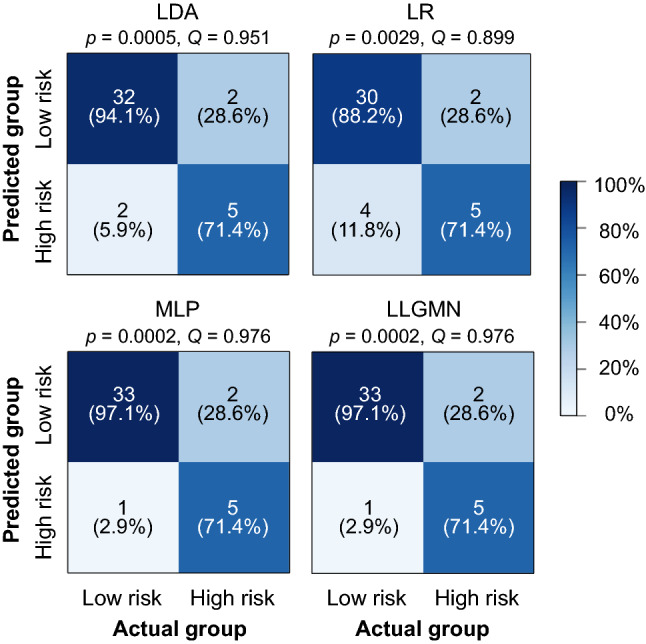
Confusion matrices summarizing the classification results by the four classifiers. Statistical test results based on Fisher’s exact test (*p*-value) and Yule’s coefficients of association (Yule’s *Q*) are also shown.

Four classifiers, LDA, LR, MLP, and LLGMN, were trained using the seven selected features as predictors. The performance of the MLP and LLGMN depends on the initial state of their random weights. Thus, hyperparameter tuning was repeatedly performed using a nested leave-one-out procedure 10 times, for both the MLP and LLGMN. After the hyperparameter tuning, the average number of nodes in the hidden layer of the MLP was 11.39 (SD = 2.0), while the average number of components in the LLGMN was 2.59 (SD = 1.43). Below, we describe and discuss the performances of the MLP and LLGMN classifiers with the highest F1 score and the highest area under the precision-recall curve (AUC-PR). The confusion matrices generated by the four classifiers are shown in Fig. 5. Fisher’s exact test on the data revealed significant associations between classifications made by each of the four classifiers and grouping by the M-CHAT score ($p < 0.003$). Yule’s coefficients of the associations were above 0.85. The performance indicators evaluated using the leave-one-out procedure are summarized in Table 3.

**Table 3:** Sensitivity, Specificity, F1 score and AUCs of the four classifiers.

Model	Performance metrics				
	Sensitivity	Specificity	F1 score	ROC-AUC	ROC-PR
LDA	0.714	0.941	0.714	0.874	0.649
LR	0.714	0.882	0.625	0.866	0.590
MLP	0.714	**0.971**	**0.769**	**0.903**	**0.688**
LLGMN	0.714	**0.971**	**0.769**	0.824	0.624

Largest value for each metric is highlighted in bold.

## Discussion

The present study investigated the relationships between automatically extracted features of spontaneous bodily movements of 4-month-old infants and their M-CHAT scores at 18 months of age. Infants at risk for ASD showed atypical characteristics for a variety of bodily movement features at 4 months of age. Further, four classifiers with bodily movement features as predictors succeeded in classifying infants with low and high ASD-like behaviours. Taken together, these findings indicate that bodily movement patterns at 4 months of age can inform about ASD-like behavioural tendencies at 18 months of age.

A close look at group differences of bodily movement patterns reveals that the bodily motion patterns of infants with high M-CHAT scores are characterised mainly by (i) reduced frequency and strength of lower limb motion, and (ii) higher central frequency and larger standard deviation of body center movement along medial-lateral axis.

It is generally known that children with ASD exhibit atypical patterns of motor control, such as clumsiness, reduced muscle tone, and poor motor coordination^46–48^. These types of motoric atypicality potentially lead to reduced amplitude and frequency of limb motion and poor coordination of limb movements, as observed in the present study. Infants in the low-risk group exhibited lower central frequency than their counterparts in the high-risk group in lateral fluctuation of body center velocity. Three to six months after birth is the period during which typically developing infants start to acquire the ability to roll over from supine to prone position^12,49^. This type of gross bodily movement of rolling, or any attempts by infants to make rolling movement, probably expresses itself as power increase within relatively low-frequency range along the left-right axis. Interestingly, a seminal study by Teitelbaum *et al.*^12^ reported atypicality in or lack of rolling movement in infants later diagnosed with ASD. Thus, the emergence of rolling movement may have led to power increase in the low-frequency range in the low-risk group compared to the high-risk group.

In addition to developmental delay in rolling motion, infants at risk for ASD show poorer posture control around the midline axis compared to infants without ASD^50,51^, which must reduce gross bodily movement along the left-right axis further in the high-risk group. Lack of gross bodily movement along the medial-lateral axis has led to absence of prominent frequency components in the high-risk group, which in turn explains the diffused pattern of power distribution in this group. High central frequency in this group probably reflects the jerky pattern of movements often observed in ASD^52,53^.

Most items of the M-CHAT concern the development of socio-cognitive functions, such as the theory of mind and joint attention. Thus, the present findings nicely dovetail with the observations by Bhat *et al.* that motor delay at 6 months of age is associated with delays in social communication at 18 months of age in infants with high risk of ASD^54^. Considering these, one might be tempted to assume that features of spontaneous bodily movement, as quantified in the present study, are linked to later social development. However, it is premature to draw such a conclusion because impairments in motor and socio-cognitive functions might be connected to the atypicality of different sub-regions of identical neural structures in the autistic brain^20^.

Several studies have found that early signs of autistic traits can be detected well before the diagnosis of ASD^6–10^. Consistent with these observations, we succeeded in discriminating infants with high ASD risk from those with low ASD risk, using linear and non-linear classifiers. Overall, the four classifiers exhibited comparable performance, supporting the robustness of our findings that the bodily movement patterns at 4 months of age contain sufficient amount of information for predicting the emergence of ASD-like behaviour at 18 months of age. At the same time, the performance of the two non-linear classifiers was numerically superior to that of the linear classifiers. Astonishingly, the AUC-ROC for the MLP classifier was above 0.9, indicating the possibility that non-linear classification based on bodily movement features can achieve classification performance that is acceptable even in clinical settings.

The present findings provide preliminary results that lead to the establishment of objective markers for the early detection of autistic tendencies in children. A recent surge of studies on digital phenotyping of ASD^16,55,56^ raised the possibility that behavioural features, quantified using low-cost non-invasive sensors, could provide reliable clues for detecting children and adults at risk for ASD. In a closely related study, Anzulewicz *et al.* succeeded in classifying children with ASD from their neurotypical counterparts by submitting finger movement patterns captured by touch sensors in tablets to machine learning-based analysis^55^. A similar attempt was also reported by Ardalan *et al.*^56^ (for a brief review, see^57^). Several other studies that applied semi-automated analysis to infants’ videos revealed differential patterns of posture and spontaneous bodily motion, such as reduced variability and increased acceleration, in infants later diagnosed as neurodevelopmental disorders^28,29^. The current research extends the achievements of these previous studies by raising the possibility that atypicality in bodily movement patterns in infancy could be used for predicting later emergence of ASD-like tendencies during toddlerhood. A series of large-scale clinical studies have shown that ASD can be predicted based on the developmental patterns of brain surface structures^58^ and resting state brain activation^59^ measured using magnetic resonance imaging during toddlerhood. It would be of great interest to determine whether comparable performance on the early diagnosis of ASD can be achieved using the digital phenotyping technique adopted in the present study.

## Conclusion

In this study, we evaluated the ASD risk in 18-month-old infants based on their spontaneous bodily movements at 4 months of age captured by markerless video analysis. In the experiments, a group comparison of movement features calculated from the video images was conducted for 34 infants in the low-risk group and seven infants in the high-risk group. As a result, 14 features showed the difference between the two groups, suggesting that the spontaneous movements at 4 months of age are related to ASD risk at 18 months of age. The classification of ASD risk showed high performance for both linear and non-linear classifiers. Therefore, the risk of ASD at 18 months of age can be discriminated from spontaneous movements at 4 months of age.

The current study had some limitations. First and most importantly, we relied solely on M-CHAT in the evaluation of ASD risk in infants, which casts some doubts on the reliability of ASD risk evaluation in the present study. M-CHAT is a widely used instrument for primary screening of ASD, but is often criticized for its relatively low sensitivity and positive predictive value^60,61^. For example, Stenberg *et al.*^62^ reported that only one-third of infants, who later received the diagnosis of ASD, scored above the cutoff at 18 months old. In addition, we did not carry out follow-up interviews by clinicians^63^, which must have increased the false-positive rate. To address this concern, a stringent and thorough evaluation of ASD symptoms, that uses more reliable test battery as the Autism Diagnostic Observation Schedule (ADOS), must be integrated into risk evaluation in the future study. Second, motor impairment is often observed in other neurological and psychiatric conditions. As such, several researchers questioned the specificity of atypical development of motor function as an early sign of ASD^13,47^. Third, we are not fully confident that bodily movement patterns are linked to ASD, or to the emergence of autism-like behaviors on the sub-clinical level. To address these limitations, the usefulness of our system for early screening of children with ASD should be tested in a long-term study with a prospective design that would recruit children genetically at high risk for ASD. Fourth, although we only conducted analyses on the overall M-CHAT score, the M-CHAT includes sub-items such as perceptual and repetitive behaviors as well as social behaviors. In the future, we plan to explore the relationship between these individual items and motor characteristics in more detail.

## Supplementary Information

Supplementary Information.

## Data Availability

The datasets analyzed during the current study are available from the corresponding author upon reasonable request.
