# Supplementary material for: Prediction of autistic tendencies at 18 months of age via markerless video analysis of spontaneous body movements in 4-month-old infants
Source: Sci Rep. 2022 Oct 27;12:18045. doi: 10.1038/s41598-022-21308-y (PMC9614013; doi:10.1038/s41598-022-21308-y)
Supplement: Supplementary file 1 — Supplementary Information. [file 41598_2022_21308_MOESM1_ESM.pdf]

Supplementary Materials for

Prediction of autistic tendencies at 18 months of age via markerless video analysis of spontaneous body movements in 4-month-old infants

Hirokazu Doi[1], Naoya Iijima[2], Akira Furui[3], Zu Soh[3], Rikuya Yonei[4], Kazuyuki Shinohara[4], Mayuko Iriguchi[4], Koji Shimatani[5], and Toshio Tsuji[3,*]

[1]Department of Science and Engineering, Kokushikan University, Japan.
[2]Graduate School of Engineering, Hiroshima University, Japan.
[3]Graduate School of Advanced Science and Engineering, Hiroshima University, Japan.
[4]School of Engineering, Hiroshima University, Japan.
[5]Graduate School of Biomedical Sciences, Nagasaki University, Japan.
[6]Faculty of Health and Welfare, Prefectural University of Hiroshima, Japan.

* tsuji-c@bsys.hiroshima-u.ac.jp

The PDF file includes:

Supplementary Texts
Supplementary Tables S1–S3
Supplementary Figure S1

Supplementary Table S1: Mean scores of questionnaires sent to mother-infant pairs, excluding M-CHAT. Standard deviations are in parentheses; statistics W and p values obtained from between-group comparisons by the Brunner-Munzel test are also shown.

	Score			
Questionnaire	High risk	Low risk	W	p-value
**4 months**				
CESD	9.429 (3.505)	5.029 (5.595)	3.766	1.611×10^{-3}
EPDS	3.429 (3.207)	2.765 (3.358)	0.493	0.636
Bonding scale	3.714 (2.812)	1.147 (1.987)	1.972	0.090
KIDS	46.571 (7.480)	48.824 (8.193)	-0.805	0.440
**18 months**				
CESD	14.429 (3.919)	7.441 (7.287)	4.706	3.417×10^{-5}
Bonding scale	4.143 (4.018)	2.147 (2.311)	0.801	0.451
KIDS	48.571 (14.444)	64.059 (11.040)	-3.537	6.459×10^{-3}

Supplementary Table S2: List of items in the Japanese version of M-CHAT

Item no.	M-CHAT item	Critical
1	Enjoys being swung	
2	Interest in other children	✓
3	Climbs up stairs	
4	Enjoys peek-a-boo	
5	Pretend play	
6	Imperative pointing	✓
7	Declarative pointing	✓
8	Functional play	
9	Brings objects to show	✓
10	Eye contact	
11	Oversensitive to noise	
12	Responds to smile	
13	Imitation of action	✓
14	Responds to name	✓
15	Point following	✓
16	Walking	
17	Gaze-following	
18	Unusual finger movement	
19	Gaining parent's attention	
20	Wondering hearing	✓
21	Understands what is said	✓
22	Stares at nothing	
23	Social reference	✓

Supplementary Texts: Definitions of Bodily Movement Features

Movement Magnitude

Movement frequency (I_1)

This feature is defined as the proportion of the number of frames with bodily movement between the first and S-th sub-segments using the following equations:

$$ {}^{(A_k)}I_1 = \frac{100}{L} \sum_{s=1}^{S} \sum_{l=1}^{L_s} {}^{(A_k)}\kappa_l, \tag{1} $$

$$ {}^{(A_k)}\kappa_l = \begin{cases} 1 & ({}^{(A_k)}M_l \geq M_{\mathrm{th}}) \\ 0 & ({}^{(A_k)}M_l < M_{\mathrm{th}}) \end{cases} \tag{2} $$

where L is the total number of frames in each sub-segment, and M_{th} is the threshold for determining whether movement has occurred. In this paper, we set $M_{\mathrm{th}} = 0.005$.

Movement strength (I_2)

This feature is defined as the magnitude of movement per unit time between the first and S-th segments using the following equations:

$$ {}^{(A_k)}I_2 = \frac{1}{L'} \sum_{s=1}^{S} \sum_{l=1}^{L_s} {}^{(A_k)}\nu_l, \tag{3} $$

$$ {}^{(A_k)}\nu_l = \begin{cases} {}^{(A_k)}M_l & ({}^{(A_k)}M_l \geq M_{\mathrm{th}}) \\ 0 & ({}^{(A_k)}M_l < M_{\mathrm{th}}) \end{cases} \tag{4} $$

where L' is the total number of frames in which ${}^{(A_k)}M_l$ is M_{th} or more between the first and S-th segments.

Movement count (I_3)

This feature is defined as the number of continuous movement detections, divided by the total number of frames L:

$$ {}^{(A_k)}I_3 = \frac{1}{L} \sum_{s=1}^{S} {}^{(A_k)}Q_s, \tag{5} $$

where ${}^{(A_k)}Q_s$ represents the number of movements detected in the s-th video sub-segment. A movement is detected when ${}^{(A_k)}M_l$ becomes ${}^{(A_k)}M_l \geq M_{\mathrm{th}}$ once and then falls below M_{th} again.

Movement Balance

Ratio of Movement Frequency (I_4)

This feature is defined as the ratio of the movement frequency between the bodily regions A_{k_1} and A_{k_2}:

$$^{(A_{k_1},\, A_{k_2})}I_4 = \frac{^{(A_{k_1})}I_1}{^{(A_{k_2})}I_1}. \tag{6}$$

Note that if $^{(A_{k_2})}I_1 = 0$, then $^{(A_{k_1}, A_{k_2})}I_4 = 0$.

Ratio of Movement Strength (I_5)

This feature is defined as the ratio of the movement strength between the bodily regions A_{k_1} and A_{k_2}:

$$^{(A_{k_1},\, A_{k_2})}I_5 = \frac{^{(A_{k_1})}I_2}{^{(A_{k_2})}I_2}. \tag{7}$$

Note that if $^{(A_{k_2})}I_2 = 0$, then $^{(A_{k_1}, A_{k_2})}I_5 = 0$.

Movement Coordination (I_6)

Movement coordination between the bodily regions A_{k_1} and A_{k_2} is calculated as the correlation coefficient between the time-series data of $^{(A_{k_1})}M_l$ and $^{(A_{k_2})}M_l$. Correlation coefficients were computed within sliding temporal windows with a length of 300 frames. The stride of the sliding window was one frame. Correlation coefficients were then averaged to yield a single feature of the movement coordination.

Movement Rhythm

Central Frequency (I_7) and Second Moment around Central Frequency (I_8) of Motor Alteration

The time-series data of $^{(A_k)}M_l$ of each video sub-segment were analyzed in the frequency domain. Within each video sub-segment, the time-series data within a sliding window with the length of 128 frames were subjected to the fast Fourier transformation. The stride of the moving window was one frame. Then, the average power spectrum density (PSD), $P(f)$, was computed by grand-averaging PSDs across all the sliding widows in all the video sub-segments. Based on $P(f)$, central frequency (F_{cntr}) and second moment around the central frequency

(D_{cntr}) were computed according to the following equations:

$$F_{\mathrm{cntr}} = \frac{\sum_{f=0}^{f_{\max}} f P(f)}{\sum_{f=0}^{f_{\max}} P(f)}, \tag{8}$$

$$D_{\mathrm{cntr}} = \sqrt{\frac{\sum_{f=0}^{f_{\max}} P(f)(f - F_{\mathrm{cntr}})^2}{\sum_{f=0}^{f_{\max}} P(f)}}, \tag{9}$$

where $f_{\max} = 15$ Hz is the maximal frequency range for analysis. F_{cntr} and D_{cntr}, computed on the basis of $P(f)$, correspond to ${}^{(A_k)}I_7$ and ${}^{(A_k)}I_8$, respectively.

Central Frequency (I_{9x}, I_{9y}) and Second Moment around Central Frequency (I_{10x}, I_{10y}) of Body Center Velocity

The time-series data of the body center velocity along the x- (top-bottom) and y- (left-right) axes were subjected to the fast Fourier transformation. Based on the resulting PSD, the central frequency and the second moment around the central frequency were computed in essentially the same manner as ${}^{(A_k)}I_7$ and ${}^{(A_k)}I_8$.

Central Frequency (I_{11x}, I_{11y}) and Second Moment around Central Frequency (I_{12x}, I_{12y}) of Body Center Fluctuation

The time-series data of body center fluctuation along the x- (top-bottom) and y- (left-right) axes were subjected to the fast Fourier transformation. Based on the resulting PSD, the central frequency and the second moment around the central frequency were computed in essentially the same manner as ${}^{(A_k)}I_7$ and ${}^{(A_k)}I_8$.

Movement of the Body Center

Variation in the Body Center Velocity (I_{13})

The average absolute values of the body center velocity along the x- ($G^v_{l,x}$) and y-axes ($G^v_{l,y}$) were computed using the following equations:

$$\left({}^{(A_7)}I_{13_x}, {}^{(A_7)}I_{13_y}\right) \tag{10}$$

$$= \left(\frac{1}{L}\sum_{s=1}^{S}\sum_{l=1}^{L_s}\left|G^{\mathrm{v}}_{l,x}\right|,\ \frac{1}{L}\sum_{s=1}^{S}\sum_{l=1}^{L_s}\left|G^{\mathrm{v}}_{l,y}\right|\right). \tag{11}$$

Standard Deviation of Body Center Fluctuation (I_{14})

The standard deviations of the body center fluctuations along the x- ($G^d_{l,x}$) and y- axis ($G^d_{l,y}$) within the sliding temporal window of length L_{g} are calculated as

follows:

$$\sigma_j = \sqrt{\frac{1}{L_\mathrm{g}} \sum_{l=1}^{L_\mathrm{g}} \left(G_{l,j}^\mathrm{d} - \overline{G}_j^\mathrm{d} \right)^2}, \tag{12}$$

where $j \in \{x, y\}$, $L_\mathrm{g} = 300$ frames, and $\overline{G}_j^\mathrm{d}$ represents the average value of $G_{l,j}^\mathrm{d}$ within the window. The σ_j is calculated in all video sub-segments, in the same way; then, I_{14_i} is calculated by averaging all σ_j.

Area of Body Center Excursion (I_{15})

This feature represents the area surrounded by the outermost circumference of the trajectory of the body center excursion. First, in the sliding temporal window of length $L_\mathrm{g} = 300$ frames, the outermost circumference of the trajectory of $(G_x^\mathrm{d}, G_y^\mathrm{d})$ are arranged in a clockwise direction based on the method proposed by Kim *et al.* [1] and defined as $(G_{b,x}^\mathrm{d},\ G_{b,y}^\mathrm{d})$ ($b = 1, 2, \ldots, B$; B is the total number of points of the outermost circumference). The area surrounded by the outermost circumference is then calculated using the following equation:

$$\frac{1}{2} \sum_{b=1}^{B} \left| G_{b,x}^\mathrm{d} G_{b+1,y}^\mathrm{d} - G_{b,y}^\mathrm{d} G_{b+1,x}^\mathrm{d} \right|. \tag{13}$$

Note that if $b = B$, then $b + 1 = 1$. This calculation is performed continuously, sliding the window by one frame. The areas are calculated in all video sub-segments, and the average of all obtained areas is defined as the feature ${}^{(A_9)}I_{15}$.

References

[1] Kim, G., Ferdjallah, M., Harris, G. F., Fast computational analysis of sway area using center of pressure data in normal children and children with cerebral palsy, *Am. J. Biomed. Sci.*, **1**, 364–372, (2009).

Supplementary Texts: Analysis of the relationship between bodily movement features and ordinal scores of M-CHAT items

Methods

To investigate the relationship between motor features at 4 months and ordinal scores of M-CHAT items at 18 months, regression analysis based on a generalized linear model (GLM) was performed. The subjects were 41 infants (seven in ASD high-risk group and 34 in ASD low-risk group) as in the main text.

The objective variable of the GLM was the ordinal score of the M-CHAT items at 18 months (the number of failed items on the M-CHAT), and the explanatory variable was the 26 motor features obtained from the video at 4 months. Because the ordinal scores of the M-CHAT items can be regarded as count data with an upper limit, a binomial distribution was set for the GLM error structure, and a logit function was selected for the link function. Forward-backward stepwise model selection based on Akaike's information criterion (AIC) was performed to search for the best model for predicting the ordinal scores. The prediction accuracy of the selected model was evaluated based on leave-one-out cross-validation.

Results and discussion

Supplementary Table S3 shows the information on the best regression model obtained from the stepwise analysis. In total, ten features were selected as the best features for predicting the ordinal scores of the M-CHAT items. The results of the model accuracy evaluation by cross-validation showed a mean absolute error between the predicted and true scores of 0.459. The relationship between the true and predicted scores is shown in Supplementary Figure S1. A significant positive correlation was found between both scores ($r = 0.6214; p < 0.0001$)

Effective movement features were almost consistent whether the ASD high- and low-risk groups were compared dichotomously based on a cutoff criterion or the M-CHAT score itself was regressed. Specifically, among ten features selected by this analysis, the following eight features showed significant differences between the ASD high- and low-risk groups (see main text): ${}^{(A_6)}I_2$, ${}^{(A_7)}I_{10y}$, ${}^{(A_7)}I_{11x}$, ${}^{(A_7)}I_{11y}$, ${}^{(A_7)}I_{12y}$, ${}^{(A_7)}I_{13y}$, ${}^{(A_7)}I_{14x}$, and ${}^{(A_7)}I_{15}$. These results support the validity of the proposed video-based movement analysis method for assessing ASD-like behaviors in infants.

Supplementary Table S3: Features selected by the stepwise analysis based on a generalized linear model.

Features	Estimate	Std. Error	z-value	p-value
(Intercept)	-0.654	4.944	-0.132	0.894
${}^{(A_6)}I_2$	-264.424	94.0	-2.813	4.908×10^{-3}
${}^{(A_7)}I_{11_x}$	20.277	7.297	2.779	5.457×10^{-3}
${}^{(A_7)}I_{11_y}$	-37.753	12.295	-3.071	2.136×10^{-3}
${}^{(A_7)}I_{12_y}$	36.693	10.859	3.379	7.270×10^{-4}
${}^{(A_7)}I_{13_y}$	221.799	66.228	3.349	8.110×10^{-4}
${}^{(A_7)}I_{14_x}$	210.40	136.119	1.546	0.122
${}^{(A_7)}I_{15}$	-3015.140	974.782	-3.093	1.980×10^{-3}
${}^{(A_7)}I_{9_x}$	-18.689	6.284	-2.974	2.939×10^{-3}
${}^{(A_5,A_6)}I_6$	23.991	7.717	3.109	1.878×10^{-3}
${}^{(A_7)}I_{10_y}$	12.994	5.944	2.186	0.0288

${}^{(A_6)}I_2$: Movement strength (lower body). ${}^{(A_7)}I_{11_x}$: Center frequency of body center fluctuations (x axis). ${}^{(A_7)}I_{11_y}$: Center frequency of body center fluctuations (y axis). ${}^{(A_7)}I_{12_y}$: Second moment around ${}^{(A_7)}I_{11_y}$. ${}^{(A_7)}I_{13_y}$: Variations in body center velocity (y axis). ${}^{(A_7)}I_{14_x}$: Variations in body center fluctuations (x axis). ${}^{(A_7)}I_{15}$: Area of body center excursion. ${}^{(A_7)}I_{9_x}$: Center frequency of body center velocity (x axis). ${}^{(A_5,A_6)}I_6$: Symmetry in upper and lower body. ${}^{(A_7)}I_{10_y}$: Second moment around ${}^{(A_7)}I_{9_y}$.

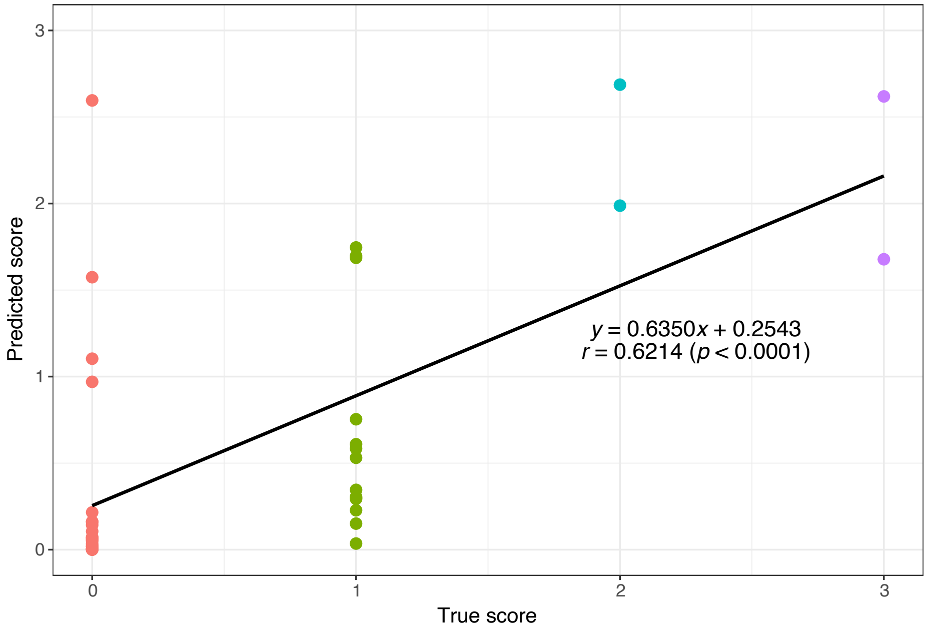

Supplementary Figure S1: Relationship between the true and predicted values of ordinal scoring of M-CHAT items. The solid black line and the equation show the results of fitting a linear regression model to the relationship. Pearson's correlation coefficient (Pearson's r) and the results of the test of no correlation are also shown.
